# A Neuroscientific and Cognitive Literary Approach to the Treatment of Time in Calderón’s *Autos sacramentales*

**DOI:** 10.3389/fnint.2022.780701

**Published:** 2022-03-28

**Authors:** Alejandra Juno Rodríguez Villar

**Affiliations:** Hanover College, Hanover, IN, United States

**Keywords:** Calderón de la Barca, *autos sacramentales*, cognition, theatre, time, Spanish, literature, Early Modern

## Abstract

Time processing is a fundamental subject in cognitive sciences and neuroscience. Current research is deepening how our brains process time, revealing its essential role in human functionality and survival. In his *autos sacramentales*, Early Modern Spanish playwright Pedro Calderón de la Barca portrays the relationships between human inner workings and the Christian concept of time. These plays portray the experience of the present, the perception of the flow of time, the measure of time raging from seconds to eternity, and the mental travel necessary to inhabit the past and future with the help of memory and imagination. Calderón explores how the dramatic form can portray all these temporal phenomena and how that portrait of time can constrain the dramatic structure. The different parts of the brain in charge of executive decisions, projections, memories, computation, and calibration are the basis that leads these characters to make the choices that will take them to the future they have cast for themselves. This paper analyzes how the processes that Calderón ascribed to the soul of his characters in the 17th century relate to ongoing cognitive and neuroscientific findings.

*“The longer you can look back, the farther you can look forward.”*

*Winston Churchill^1^*

## Introduction

In 17th century Spain, the Aristotelian precept “art imitates nature”^2^ is the rule, and theater, the most mimetic mirror of nature among the arts, mimics the experience of time. One of the dramatic forms in which this reflection is profuse is the Spanish genre *autos sacramentales*. In his *autos*, the Spanish playwright Calderón de la Barca (1677) reflects on how time defines human existence and shows, simultaneously, how the Christian understanding of time can function as a constraint on the dramatic structure. The playwright’s diverse productions contain many explicit references to time, but time ontology and its effects on human existence are intrinsic to the structure and plot settings of these *autos sacramentales*. This article reflects on Calderón’s take on time and how this was influenced by his cultural environment. Subsequentially, this essay, drawing from cognitive and neuroscience research, analyzes how time is organically measured, grasped, and felt by these characters and in these dramatic structures, and finally, how that embodied experience of time^3^, from emotions to movement, is a fundamental component of this genre.

## The Cognition of Time as a Fundamental Topic in Spanish Early Modern Literature

Inquiry about time has been a constant in Western thought since antiquity. The topic has been traditionally examined mainly from the perspective of Philosophy and Physics. Starting with Parmenides (5th c. BCE), who asserted that time is an illusion, and ranging from the famous St. Augustine’s (2021b) *Confessions* (4th c. CE), in whose Book IX, reflects on the nature of time, to the Kantian transcendental aesthetic aprioristic concept of time (18th c. CE), no period has failed to contribute to the discussion. In the past century, cognitive science and neuroscience disciplines have joined the debate, adding psychological and physiological standpoints to the study of the experience of time.

Far from having a unified account about what time is or how it operates, the debate continues. According to Western understanding, nothing happens without extension in time^4^. This ubiquity adds complexity due to the countless ways in which we can approach time. From the ability to consciously or unconsciously grasp time to estimate durations that go from zeptoseconds to lifetimes, to its perceived speeding up or slowing down, prediction or postdiction, mental time travel through memory and imagination, the starting points are abundant.

Time is still the great unknown, and, like other disciplines, neuroscience has not come to a single explanation of how our bodies measure or perceive time. At this point, there are two main theories. The first one, the *dedicated model*, proposes that dedicated neural circuits in the brain process time operations ranging from milliseconds to seconds^5^. This means that the brain would have distinctive and specific mechanisms to process time. The second theory, the *intrinsic model*, argues that the measure of time is an emergent property of regular neural processes and their inherent dynamics^6^. In the intrinsic model, there is not a specific part of the brain dedicated to processing time; on the contrary it is the neurons’ dynamic behavior which encompasses the timing to carry out physical actions. Regarding this, Buonomano and Laje (2010) have proposed the concept of *population clocks*, a model in which, “a given point in time is represented by a unique spatial pattern of activity within a neural network. Distinct patterns of activity in the network unfold over time.” In other words, the brain would encode time in dynamic patterns of neural activity.

As is to be expected, Early Modern Spanish Literature is very far from the contemporary discussions of these two models. Nevertheless, it chooses the conscious perception of time as one of its essential topics. This period of Spanish literature is split chronologically between the 16th and 17th centuries, or, using traditional historiographical terms, between the Renaissance and the Baroque. This period evolves from an early vital optimism to a crepuscular pessimism. This evolution is of critical importance, considering both mindsets are appraisals related to the future. Deep into the 17th century, the tropes of *carpe diem, tempus fugit*, and *memento mori^7^* will dramatically permeate Spanish cultural production. Due to the Early Modern scientific revolution, it becomes easier to measure time more accurately, and the rise of capitalism turns it into a valuable good. The intensification of individualism and the high mortality rates increase the feeling of time fleeing away under the omnipresent remembrance of death, which influences several Baroque Spanish writers to perceive this period as decaying.

Paradoxically in this age, Spain gives birth to a dramatic genre that will be the epitome of optimism^8^: *autos sacramentales*, a genre of religious plays that evolved from medieval moral plays, many times set as *psychomachias* or interaction between the aspects of the mind, and developed in the light of the Catholic Counter-Reformation. Standing out among the most belligerent points between Catholics and Protestants in this period is the concept of free will. While Catholics believe in human choice as an instrument to reach salvation, Protestant reformists plead that people are predestined to salvation or damnation even before birth. Catholics believe in a more open future, to a great extent decided by the person through their decisions, and in which providence will very likely grant forgiveness.

### *Autos sacramentales* as a Human Measurement of Time

*Autos sacramentales* tell the story of Humankind’s redemption, following the conventional arch (grace, fall, redeeming sacrifice of Christ), and they often end with an exaltation of the Eucharist. They simultaneously recount the History of Man from the present, dating back to the beginning of time, and projecting to the end of days. The *autos sacramentales* penned by Calderón de la Barca (1677) are considered the summit of this genre, and in his words, they are *performed sermons^9^*. They are developed in just one act encompassing 1000–2000 verses in the form of allegories. An allegory is a system of intertwined metaphors in which abstractions are often anthropomorphized as characters. The allegorical structure allows the play to tell several stories that overlap in one plot, playing with a text and several subtexts. *Autos* have an *asunto*, the timeless idea of human redemption, and an *argumento*, the storyline through which the *asunto* takes form. These *argumentos* are drawn from the Bible, Catholic doctrine, hagiographies, mythology, or history and Calderón has his characters refer to this idea explicitly in his *autos^10^*. Lastly, *autos sacramentales* share with the mass the endeavor of *performing* certain mysteries of the Catholic tradition, like the Eucharist, embodiment of the redemption, and they do this *in community*.

This genre is historical as it portrays humankind’s history from a doctrinal standpoint. In this vein, Barbara Kurtz points out, “In focusing intense dramatic attention on history, Calderón often merely echoes centuries of Christian speculation and Catholic doctrine. This theory and doctrine suggest that Christianity veritably begins *with* history and that Christian theology begins *as* history” Kurtz (1991, p. 122, emphasis in the original). Because of this, Calderón’s *autos* usually have fallen into the consideration of the timeless: “The Eucharist, the ‘symbolum unitatis,’ gathers into its own timeless unity the whole of mankind’s spiritual history and destiny” (Parker, 1968, p. 76). This opinion stems from the fact that the very same matter of the *autos* makes them achronic: “The fight between good and evil never stops, and the redeeming capability of the Eucharist is eternal. The *auto* might be the dramatic genre that most systematically treats time as something eternal”^11^ (Spang, 1997, p. 492). However, as several scholars have argued, *autos sacramentales* are also embedded in their specific spatial and temporal coordinates, occasionally referring to events in secular human history (Greer, 1997a,b; Paterson, 1997). The passage of time is often the background of Calderón’s *autos*; his characters reflect on it, and Time even appears as a character in some of the plays^12^.

## Physics, Neuroscience, and Doctrinal Overlaps on the Concept of Time

### Time as Change

Aristotle (2021b) relates time to change, stating that there is no time without change, neither change without time (*Phys.* IV). Calderón also insists on this symbiosis: “>Quieìn puede dudar del Tiempo ser continuas las mudanzas?^13^” (Who doubts that change is a constant in time?) If time is related to change, it is reasonable to think that the perception of time must disappear in the absence of change. This lack of change refers to the literal timelessness of the *autos*, timelessness that only takes place in states of sanctifying grace (in its original form at the Paradise stage). Calderón explicitly develops this idea in Calderón de la Barca (2000b) *El veneno y la triaca* (The Poison and the Antidote). Until the commission of the original sin by a Princess (a metaphor for humankind), everything remains unchanged and, therefore, timeless. This timelessness is exemplified by the four personified seasons (the Princess’ maids), acting as if they were all of them, springtime. Until the fatal error, the Princess lives in a constant spring “porque aquí son todos/Primavera solamente” (vv. 686–687) (all seasons are here Spring), indicating thus the suspension of the passage of time (Calderón de la Barca, 2000b, p. 88). Calderón uses the idea of a never-changing spring in several other *autos^14^* to delve into the time-change binomial. Another way Calderón conceptualizes the never-changing setting is as an eternal garden. It will be necessary to have an action, a change, to set time in motion, to set history in motion, and this is what the original sin will do^15^. In Calderón de la Barca (2000b) *El veneno y la triaca*, Calderón entrusts the character of Innocence with this idea: “Sin tiempo nada pasó” (nothing happens outside time, v. 895). This initial setting of the everlasting spring emphasizes the lack of change and, therefore, God’s inherent timelessness. But Calderón knows that drama is conflict, and to arise, conflict needs change, in this case, more than ever, a unit of disorder.

### The Arrow of Time

To understand these plays’ ethos, we must pay attention to one of these texts’ most salient characteristics: their insistence on the concept of linear time. According to Christian doctrine, to the eyes of mortals, time has a beginning and an end. From the Creation to the Final Judgment, events are located in a line advancing from the past through the present and into the future without repetition. They are unique in themselves in their ordinal quantitative condition.

Christian doctrine divides historical periods into three ages that follow each other consequentially: the Natural Law (the one that every human being has written in his heart, the Paradise), the Written Law (The Old Testament), and the Law of Grace (The New Testament).^16^ Each of these periods is caused by and follows its predecessor (expulsion from Paradise, advent of the Messiah). While the first two laws are limited in time with a specific beginning and ending, the third one, Christian redemption, has an indefinite duration. It will be effective until the end of times, becoming the present and the future for the audience. The succession of these three periods is coherent with the linear, teleological time in which history flows from point A to point B in a progressive dynamic.

This historical sequencing seems ever present because, although not all cultures understand time as an autonomous, abstract conceptual domain independent of things, or something in which things happen, all of them have concepts for ordering and sequences of events (Sinha et al., 2011). This might be explained by the fact that the brain also orders events sequentially, contributing to the perception of the arrow of time. Neuroscience has reliably identified the brain regions involved in the temporal sequence of representations. This does not mean neurons compute time, or that the brain generates a representation of temporal sequences (Friston and Buzsáki, 2016). Rather, this sequential organization is generated by the hippocampus through a “sequential activity of neuronal assemblies and their internally defined rate of change without resorting to the concept of space or time” (Buzsáki and Tingley, 2018). These ordinal sequences in neuronal trajectories represent past, present, and future, explaining why postdiction, imagination, and prediction overlap, as fMRI scans have shown (Schacter et al., 2007). This overlapping happens with activation of lateral and medial prefrontal cortices, precuneus, posterior cingulate, and retrosplenial cortices, lateral and medial temporal areas, including the parahippocampal cortex, and the hippocampus (Hassabis et al., 2007a,b).

In the Christian history narrative, this sequence of historical events expands toward disorder. We depart from a highly ordered and changeless state (Eden), in which something happens (Original Sin) that unleashes a state of complete disorder and change that is never completely recomposed. That evolution occurs in the form of the traditional dramatic structure: order–breaking of order–-restoration of order, both in the big scheme of things and in each character’s story. Nevertheless, neither in Christian history nor in the narrative structure can the final order be equal to the original one.^17^ Coming back to the same order would mean we are in a time loop, a concept rejected by Christian doctrine. Not in vain, St. Augustine said: “For once Christ died for our sins; and, rising from the dead; He dieth no more.”^18^ The concept of growing disorder is ingrained in these narratives in such a way that only divine intervention can reverse this flow. This intervention does not mean that humans cannot choose the good path through their free will; it means that they need, and they will be assisted somehow by God’s grace. In the pattern of grace—sin—recovery of grace, the latter will always be granted by the advent of God in the form of Christ, Grace, Church, or Inspiration. In that sense, God represents a subversion of the physical law.

Physics conceptualizes this linearity as the arrow of time, which derives from the second law of thermodynamics. This principle explains why we perceive that time only flows in one direction, but this is only because of a boundary condition. The second law of thermodynamics does not require a highly ordered beginning point from which entropy can increase; only that entropy cannot decrease in an isolated system^19^ (therefore the intervention of an “outer” character in these plays, God.) The fact that entropy never decreases makes us infer that time can only go in one direction: toward disorder.

Entropy is a physical property also present in the brain, and it refers to a number of possible neural states. Brain Entropy (BEN), is the measure of the brain’s flexibility to process unpredictable stimuli and respond predicting outcomes in an unpredictable world. BEN has been used in the past two decades to quantify the brain’s information processing, and according to the Entropic Brain Hypothesis, the entropy of spontaneous brain activity indexes the informational richness of conscious states (Carhart-Harris, 2018). This phenomenon has also been related to intelligence (Saxe et al., 2018) and divergent thinking or creativity. FMRI scans have shown a strong positive correlation of divergent thinking with brain entropy in the left dorsal anterior cingulate cortex/pre-supplementary motor area and left dorsolateral prefrontal cortex, suggesting that creativity is closely related to the functional dynamics of the control networks involved in cognitive flexibility and inhibitory control (Shi et al., 2020). These findings link brain entropy with the creation of alternative scenarios and articulations. This ability is at the core of the traditional narrative structure, in which from a single starting point the possible derived narratives and how they are articulated are endless. This is more easily understandable when we take into consideration that “A brain capable of large variability in neural configurations, or states, will more easily understand and predict variable external events.” (Saxe et al., 2018). And not only that, if information entropy is necessary to create new stories, and the brain needs to have the flexibility of developing a more entropic neural configuration to react to external stimuli, as Owen et al. (2021) has proven, also high-level cognition during story listening is reflected in high-order dynamic correlations in neural activity patterns. Coincidentally, divergent thinking is close to the disobedience to the rules, which, on not few occasions, causes the doom of Calderón’s characters.

Despite this growing disorder, the flow of time in one direction must necessarily lead to a learning outcome both in *autos sacramentales* and Christian history. Returning to the starting point would mean the character would have learned naught and time would have been wasted; new developed neural states would have not arisen at the brain. For this reason, *autos sacramentales* account of human history reverses the arrow of time. From the original order, there is a process of entropy that later evolves toward an order. As stated earlier, this final stage in dramatic terms is called “restoration of order.” The essential matter in these texts is that restoration of order equates to the stopping of time in Christian history and theater because, in both cases it means the end. The entropy is over, and there are not more possible scenarios.

### The Cause-Effect Relation and Time

As mentioned before, the three ages in the History of Christianity happen consequentially. The ordinal representation of events in this arrow of time is also linked to cause-effect perception, a crucial aspect of these plays’ conception of reality, intimately related to the sequential ordering: cause must precede the effect. Likewise, timing is critical in how we perceive the relationship between cause and effect. Traditionally, it has been accepted that the more prolonged the time interval between two events, the smaller the associative strength between the two (Shanks and Dickinson, 1987). Psychology research has shown that the experienced event order affects people’s causal judgments when events occur in continuous time^20^ (Lagnado and Sloman, 2006). The *Intentional Binding Effect* describes the subjective compression of the time interval between a voluntary action and its external sensory consequence, reinforcing the sense of agency, or in other words, the experience of controlling action to influence events in the environment (Haggard et al., 2002). In terms of external sensory stimuli, in a matter of milliseconds, our brains perform causal inferences though a hierarchical order of spatiotemporal computations starting with early segregated unisensory representations, followed by sensory fusion in the parietal-temporal regions, and culminating as a causal inference in the frontal lobe (Cao et al., 2019). Related, research has also shown that when confronted with an object, one of the first automatic human reactions is *infer affordance*: we think of the object in terms of object and function. For this reason, there are clusters of neurons specialized in connecting the physical properties of objects to cognitive-motor programs to use these objects in the most efficient way (Maranesi et al., 2014).

As a genre about agency in which right and wrong decisions are crucial, *autos* make sure that the relationship between cause and effect is immediate. While other later narrative genres play with the distance between cause and effect to engage the audience’s attention, the objective here is to transmit a clear lesson (how to lose or reach eternal grace), often using objects with symbolic or metaphorical meaning. This relationship must be evident to the audience. Sin and loss of grace will occur sequentially in a matter of instants. In Calderón’s *autos sacramentales* we encounter a Newtonian universe often based on primary experiences with the physical-mechanical world. These experiences are often presented by the playwright through a physical cause that turns into a metaphorical/immaterial effect. A princess eating an apple and right away losing her state of grace, or the character of Soul tripping and being hold by Sin, are good examples of how Calderón sets this immediate relation because-effect. Nevertheless, this performing device is not exclusive of Calderón or this genre, but a core setting of Christian rituals, in which the infusing of grace becomes visible thanks to physical action. The Sacraments themselves are defined as the outward sign of an invisible spiritual reality^21^. To visualize this the ritual resorts to physical actions such as the pouring of the water in the baptism.

## Natural and Mechanical Time Measurements

Calderón’s life occupies almost the 17th century when the exact measure of time is part of the scientific discussion, and the playwright mentions clocks in his *autos^22^*. In Calderón de la Barca (1995a) *Andrómeda y Perseo*, the Devil calls Medusa, his assistant and representative of death and guilt, “*clock of the moments*” (v. 346); in Calderón de la Barca (1996c) *La nave del mercader*, the character Time is qualified as “*life clock*” (v. 686); and in Calderón de la Barca (2011a) *El pleito matrimonial del cuerpo y el alma*, the clock is identified with the lifetime: *“Do not derange this human clock!*” (vv. 1351–1352). Calderón also mentions the clock in Calderón de la Barca (2014a) *El divino cazador.* In this *auto*, Género Humano, Humankind, refers to his heart as a clock, which was becoming an increasingly popular metaphor (Sawday, 2007). In those verses, describing how kind was nature to him before the original sin, Humankind also mentions the air, which rules the breath in its role to guide the heart clock^23^, referring to the respiratory and circulatory systems. When the playwright uses the clock, it is as a metaphor for human experiences.

In contrast to his secular plays, where Calderón seizes upon the dramatic possibilities created by the recent invention of the clock^24^, Calderón’s occasional use of the clock in in the *autos* denotes the collision of two worlds. As Greer (1994, 203) points out, there is a *“fundamental incompatibility between two concepts of time: (1) the countryside time, the time of human passions, the time of nature, measured by the sun cycles and (2) the urban time, the time of the court and the King, measured by the clocks synchronized with the palace clock.*”^25^ The sparse use of clock-time in the *autos* situates them in the time of nature. It is a time with a profound moralizing goal that befits an event-time culture, rather than a clock-time culture (Lauer, 1981), as is prevalent in countries where the clock is used to regulate time. In event-time cultures, time is measured related to events or tasks. Tasks are organized depending on other tasks, and individuals pass from one to another when there is an inner conviction of the conclusion of a given task (Avner and Sellier, 2011). Event-time is the model used in the Christian history of time, in which events differentiate the ages of the three laws (natural, written, and law of grace), and it is the events contained within, rather than the duration, that marks each period. Event-time use is still prevalent in Southern European cultures, among others. Because time is measured according to task, tasks can have moral or affective qualities. Time is therefore beyond the clock, revealed through more abstract concepts, such as interpersonal relationships. This gives punctuality much less importance in event-time cultures, as it is devoid of transcendent meaning. In this cultural context, a task is not concluded because a clock marks so. Calderón is from this same cultural context; as such, he leans toward using natural and social time measurements in his *autos*, distancing his plots from artificial measurements.

## Perception of Time and Shaping of the Dramatic Structure

Calderón’s *autos* also contain many references to time intervals, and the use of the associated semantic lexicon is profuse. Measurements such as days, months, weeks, years, and centuries appear in the *autos*, with a predominance of years and centuries over days. Years, centuries and days are also more mentioned than weeks and months. Weeks are an arbitrary measurement not related to organic processes and do not seem to have the symbolic potential of days and long measurements. In the Gregorian Calendar, enacted by Pope Gregorius XIII in 1582 and adopted first in the Hispanic territories, months do not follow the lunar cycle, so they are not coincidental with any natural measurement. Calderón mostly uses days as units of time, and years and centuries as signs of eternity, contrasting two types of time interval.

### The Circadian Rhythm as the Measure of the Plot

When we refer to dramatic time, the Early Modern Period is under the influence of the Aristotelian unity of time, which rules that the action must occur in 24 h. Interestingly, although Aristotle was vague regarding this unit in his *Poetics*, his Renaissance Italian interpreters insisted upon it, constraining the plot’s duration to not more than one day^26^. The goal of this unit was to render a perfect imitation of nature. By the time Calderón is writing, Spanish dramatists have dismissed this unity of time, introducing temporal ellipsis in the intermissions. Despite this, the notion of the day^27^ as a complete unit is not alien to the *autos sacramentales*. Calderón uses the day as a metaphor for the entire human life. Dawn represents birth, and the sunset, death. The day measure also connects to the Christian doctrine of “*Dies Dierum*,” the day of the days, among which Sunday has a particular prevalence.^28^ In contrast, the night often portrays death and sin. Accordingly, sleeping operates in the *autos* as the halfway point between life and death; it represents a pseudo-death due to the loss of consciousness that it entails.

The goal behind the “Aristotelian” unity of time is verisimilitude; the day as a unit attempts to align verisimilitude with perception. However, as Anne Ubersfeld says: “far from historicizing theater, the unity of time theatricalizes history” (128), as it forces to compress events in a single day, events that in realistic circumstances would take more than 24 h (Ubersfeld, 1999). As *autos* are a genre that theatricalizes the entire history of humankind in a highly metaphorical way, the day, as a measure of time, befits them without the verisimilitude concern.

One of the most definite and conscious measurements of the flow of time for humans is the alternation between day and night. As we have seen, this shift has also proven itself to be a milestone in the debate about mimesis, precisely by its closeness to organic experience. The circadian cycles are an endogenous timing system with a length of 24 h in which physical, mental, and behavioral changes occur. Almost every tissue and organ possesses molecules (proteins) that mark these 24 h cycles, along with the processes by which these molecules are produced and broken down. This creates the circadian rhythm. In a sense, it is a birth and death that happens daily. These processes for producing and breaking down molecules are called transcriptional and translational feedback loops (TTFLs), and they are a crucial part of time processing. The synchronization of all these TTFLs, the peripheral clocks, is made from the master clock, the hypothalamic suprachiasmatic nucleus (SCN). The circadian cycle affects time perception (Kuriyama et al., 2003). It also marks human present-orientedness, as “today” is clearly defined by the wake-sleep cycle. This cycle explains why Renaissance theorists understood the day as a basic unit of time. The day works as a metaphor of time intervals with naturally marked beginnings and endings, or births and deaths. It is extensive, manageable, and organic enough to have served as the primary time unit since 1500 BCE, when Egyptians invented the sundial up, to the emergence of mechanical clocks.

### Infradian Intervals and the Concept of Cyclical Time

Cyclical time is impossible in the theological Christian view as we have seen; however, it is part of the human experience. *Autos sacramentales* rely on the cyclical nature of creation to intertwine the perception of time with the computation of time. Infradian measurements, those beyond 24 h, relate especially to seasons, which the human body can also measure. The pineal gland secretes melatonin by night, which marks the photoperiodic seasonal rhythms in mammals. This gland is itself controlled by the circadian rhythms ruled by the SCN. The length of the night regulates the melatonin secretion, influencing the season measurement. Astrocytes, specialized glial cells in the brain, initiate the neuronal oscillations of the SCN. The interaction of neurons and astrocytes is behind how the SCN encodes and distributes circadian and seasonal information (Hastings and Brancaccio, 2020).

Given the organic processing of seasons, it is not surprising that the playwright uses them as his preferred method of signaling the passage of time and the cyclical nature of time in human perception. In Calderón de la Barca (2010) *Los alimentos del hombre* (The Sustenance of Man), after the commission of sin, the seasons reveal to Adamo (an allegory of humankind) how he will have to deal with them from that moment on, with an apparent focus on farming and cattle, as all four characters appear with props referring to those activities. In their lines, all seasons remark on their cyclical nature.^29^

Beyond these natural rhythms there is another way to experience cyclical time^30^ in Calderón’s *autos*: characters in a state of guilt, or negative characters, namely, the Devil and his henchmen, inhabit hell, which is “perpetual confusion, eternal pain, punishment without redemption”^31^: a place without changes. The anguish never ends. It is an emotional state of “pain,” and research points out that “being in pain leads to an expansion of subjective time whereby a stronger increase in pain perception relative to non-painful stimulation leads to a stronger time-estimate distortion” (Rey et al., 2017). This slowing down of time is not related to any particular area of the brain, but it seems to be distributed in cortico-subcortical networks (Wiener et al., 2010). Imaging studies indicate that the anterior insular cortex is critical in this phenomenon, as the area that integrates pain, self-awareness, and the sense of time. Likewise, clinical depression has been proven to slow down time perception when it comes to the subjective flow of time (Thönes and Oberfeld, 2015). The characters who inhabit hell have pain that is *infinite^32^*, consistent with the fact that time has come to a stop for them in a non-changeable state. In addition, recent neurophysiological research about pain elongating subjective time, points to a common neural substrate in time estimation and the perception of the self: pain may also intensify self-awareness, which dilates the subjective duration of the pain (Rey et al., 2017). This circumstance matches well with the state of Calderón’s negative characters: they are self-centered, immersed in a continuum between pain and self-attention, while positive characters in these *autos* are devoted to others. This conception of hell is also in agreement with the phenomenon of “temporal summation,” in which by increasing the duration of the pain, the very same perception of pain (nociception) increases (Perrota et al., 2016). Infinite pain is equal to infinite time perception; likewise infinite time in punishment leads to infinite^33^ pain.

## Temporal Characterization of Dramatic Protagonists

### Zeitgebers, Interoception and Hunger

Returning to the question of the “day,” when Calderón uses social divisions of daily time, he does it around religious practices. For instance, in Calderón de la Barca (2004a) *El día mayor de los días*, the only reference to human-made time measures rolls back to the canonical measurement created in the medieval monasteries. The play recalls the religious division of the day, the calls to prayer, which are the social cues with which time is also measured: “to split apart the ascetic time devoted to prayer, to the “objective” time necessary for other activities^34^” (Perez-Magallón, 2009, 937).

These external cues are called *zeitgebers* (time-givers), a term coined in Jürgen Aschoff (1960) by the German chronobiologist, and they are essential for conscious human measurement of circadian cycles (Kalsbeek et al., 2012.) Light is the most important one, but artificial zeitgebers such as mealtimes or social activities, also influence human inner clocks. Eating, if regularly scheduled daily, marks ultradian rhythms, which are timing intervals smaller than 24 h. When these peripheral clocks, such as the one for the mealtimes, become desynchronized from the central body clock, this can result in weight gain and type 2 diabetes, at least as observed in mice (Kolbe et al., 2019). The alignment between the central body clock and zeitgebers is crucial for the maintenance of metabolic homeostasis. For instance, when mealtimes are disrupted, peripheral molecular clocks get a strong signal, possibly causing the misalignment of metabolic processes until reaching the impairment of homeostasis. Consequently, the zeitgeber of eating is crucial for synchronizing circadian rhythms with metabolism (Pickel and Sung, 2020).

In literature eating is frequently linked to *interoception* which is the body-to-brain axis of sensation concerning the state of the internal body and its visceral organs (Cameron, 2001). *Interoceptive awareness* is “the ability to identify, access, understand, and respond appropriately to the patterns of internal signals” (Price and Hooven, 2018). In recent years, the concept of interoception has gained attention concerning the emergence of the self from a cognitive standpoint. This “instrumental interoceptive inference” is related to allostatic regulation and physiological integrity and results in an embodied selfhood (Seth and Tsakiris, 2018). Other functions of interoception include energy regulation (ingestion and excretion), memory, affective and emotional experience, and the psychological sense of self (Quigley et al., 2021).

This ability to detect changes in our physiology is intimately intertwined with cognition and emotional processing. The James-Lange theory defines emotions as bodily changes, meaning that experiencing emotions is closely related to autonomic responses, such as cardiovascular and digestive activity or breathing patterns (Pollatos et al., 2005). Specifically, bodily states and emotions with high physiological arousal can increase the pulse frequency registered by the body pacemaker, and therefore the subjective duration of time can be overestimated (Wittmann and van Wassenhove, 2009.) In the same way, it has been proposed from the cognitive field that arousal and bodily signals are directly connected with subjective time awareness (Ogden et al., 2015a,b). Individuals with heightened interoceptive awareness experience more intense emotions (Wiens et al., 2000), and time perception and interoceptive self-oriented processes are connected (Pollatos et al., 2014). The processing of this awareness is nested, among other areas, in the right anterior insula, which also integrates bodily pain signals, along with self-awareness and the sense of time, according to imaging research (Pouthas et al., 2005).

The interoceptive awareness of our bodies may help us perceive time, removing from the brain the sole responsibility for this task. According to Craig, the Anterior Insular Cortex (AIC) plays a crucial role in interoception awareness, and it may well function as an indexer of feelings, which are mental states based on physical processes. This indexer would allow us to detect our emotional state moment-to-moment and mark the succession of those moments from past to future. The emotional and visceral processes share common underlying neural systems, the insular cortex and the interoceptive system (Craig, 2008, 2009). Time, or more accurately, duration, is measured by those sequential moments of awareness of the interoceptive processes.

Calderón is aware of these processes, and he portrays them by exemplifying how the body tells time through interoceptive awareness. Rather than relating interoceptive processes to the measurement of the duration of time, he instead uses them to point out the discrepancy between the internal and the external clock. This discrepancy happens in Calderón de la Barca (1997c) *Sueños hay que verdad son*, where the Breadmaker defends the schedule marked by his internal clock and not any external clock^35^. The same device is used in Calderón de la Barca (2011c) *Mística y real Babilonia*, in which the text insists on how the stomach has its own clock.

On these occasions, Calderón uses interoceptive signals of hunger and sleep to contrast the time measured by a clock. His characters support these physiological feelings as the fundamental determinants of when to do things, in a clear defense of task-based time vs. clock time. There is a reason why this type of interoception is used with this type of character and no others. Different organs have been related to different social statuses since antiquity.^36^ Traditionally, the stomach has been linked to low passions and the lower social classes, and this is true for Calderón’s characters as well. In his *autos*, it is the lower-class characters that experience hunger and cravings, which means these physiological sensations only work as time markers for such characters.

### The Human Capacity to Feel Present Time

Feeling time is also part of our interception (Di Lernia et al., 2018). But what is being measured? One of the ever-present questions about time is whether it is a property of reality or just an illusion. In the same way that the flow of time might be an illusion (Gruber and Block, 2013), our brain can also make us believe time has stopped, as happens with the phenomenon of chronostasis (Yarrow et al., 2001). Our experience of time does not prove its existence. This “illusion” seems to be a necessary condition for survival, considering that the ability to measure time and duration is key to learning processes and behavior. The traditional physics debate standpoints on this topic are *presentism* and *eternalism*. Presentism states that the only real-time is now, discarding the existence of past and future. On the other hand, eternalism states that past, present, and future are equally real and exist simultaneously.

Eternalism is a perfect way to explain the physical relationship between time and space because, in the same way one can transit over space, one can traverse over time, as “now” would only mean in what “place” of time one is located. In other words, only eternalism would allow time travel. In presentism, there is nowhere to travel.

Both theories of time are ingrained in Calderón’s *autos.* In a way, it is because these two conceptions of time exist that the *autos* can set their basic premises. For human characters, time-traveling is entirely impossible; therefore, there is no way to come back to the past to mend errors. That is the reason why correct performance in the present is critical. Conversely, time does not exist for God, or more accurately, God has no sense of past, present, or future, as for God, past, present, and future are all concentrated in a continuous present. There is only “here” and “now” for God,^37^ and God is unchanging^38^, the opposite of human experience.

Because God sees everything in the present, God knows each decision’s outcomes, which gives God and God’s adjuvants the *potestas* of trying to influence human characters toward the absolute right choice. Nevertheless, this tension between foreknowledge and free will has been one of the most discussed matters in Western philosophy history. In its religious form, this opposition will be at the heart of the most critical discussions in Early Modern Catholic Europe, with Spanish philosophers and theologians’ preeminent participation^39^.

Although eternalism is the time theory required by special relativity and favored by philosophers, humans are not equipped to perceive time this way. On the contrary, humans perceive time as tensed, as having a past, present, and future. This perception also means presentism is the most intuitive theory for time. In his well-known disquisition about time, St. Augustine accepts that none of the three divisions of time exists, as the past is not anymore, the future is not yet, and the present has no extension^40^. Nevertheless, we experience time. The illusion of these divisions is due to the *distention animi* or the soul’s projection. This projection takes place through memory and imagination, anticipating modern findings on mental time-travel. fMRI studies have shown the existence of common neural structures regarding re-experiencing the past and pre-experiencing the future. This circuitry seems to be concentrated in the medial prefrontal cortex, the posterior regions, and the medial temporal lobes (Botzung et al., 2008). These areas would integrate a common neurocognitive system, which would allow humans to mentally travel through time.

This Augustinian conception of time has been called the “Christian revolution” in understanding time, as it defends that time finds its reality in the invisible distention of the human mind, or the soul, in Augustinian terms. According to St. Augustine, what we have is our present, which is always about to die because it has no extension, although neuroscientists have proved him wrong. Nowadays, there is a time measurement for the present consciously experienced, which we call the *specious present*. The specious present happens when individuals consider their perceptions as the present, and in this present, consciousness operates. We always experience in the present. This present “spans a range of ±100 ms. This range is well-motivated both neurologically and representationally as it accommodates latency differentials in sensory processing and incorporates anticipated movement by means of efference copies of motor commands” (Droege, 2009).

In these *autos*, only Christ, by his binary nature, as God and man, can experience eternalism and presentism simultaneously, connecting humanity and divinity in the experience of time. As he will say in the form of Pan in *El divino dios Pan* (The Divine God Pan)*:*

Pan: […] fue decir que hay en miì unidas
tan desiguales distancias
como hay de humano a divino,
significaìndome en ambas:
en el semblante lo eterno
y lo caduco en las plantas^41^ (vv. 73–78).

### Time and Space and the Parietal Cortex

Eternalism and presentism are intimately related to space, defining individuals’ relationship with the flow of time. The two possibilities that humans experience are the ego-moving and the time-moving perspectives. In the ego-moving view, time is a matter across which humans transit. This perspective inspires the metaphor of human life as a journey. The individual is a *homo viator*, a traveler, and Calderón often uses this as the motive of the play in the characters’ speeches^42^. As Lakoff and Johnson (1999, p. 137)^43^ have explained, time metaphors usually draw from spatial vocabulary, and *autos* also appeal to spatial terms to measure time. However, terms purely related to time also appear, such as *breve, caduco*, *efímero, perecedero*, and the word *plazo*, as a time with a deadline^44^. In these *autos* we also find temporal adverbs like *al momento, pronto, presto, tarde*, and adjectives related to speed, such as *rápido, veloz*, *lento*, or *ligero^45^*. When this happens, the *autos* show the time-moving perspective, turning time into a being with agency linked to verbs like *llegar, correr*, and *apresurarse^46^.* Likewise, we can also find relationships between time and space in how the brain analyzes perceptions to construct reality. According to his Theory of Magnitude (ATOM), Walsh (2003a,b) has proposed that measurable entities such as time and space, among others, are processed in the parietal cortex (PC) because humans learn about the environment through motor actions. This theory claims that time and space are symmetrically related in the PC. However, fRMI has agreed with Metaphor Theory on their asymmetric relationship (Gijssels et al., 2013): temporal estimations are more influenced by non-important spatial information than vice-versa, supporting the frequent use of spatial vocabulary.

## Cognitive Processes as the Temporal Fate of Characters

### Erring the Object of Attention

Calderón shows a significant concern with time measurement, often referring to its *cómputo* (count). However, except for terms specified in the Christian canonical texts, the duration of the mentioned times is imprecise. The playwright gives time estimates when he talks about the *eternal* duration of glory and the possibility of the limited duration of the state of grace, life, or vain amusement, which is to say, predictions of duration in the future, using much more prediction postdiction. Calderón presents his negative characters intending to enjoy the vanity of the present moment “whatever it lasts.”^47^ This phrasing works as a reminder that their enjoyment has a limit and echoes the difficulty humans encounter estimating prospective durations of time, plus the uncertainty derived from human experience^48^. It is not the same to estimate the prospective time length when there is an accessible comparison, which Calderón’s characters do not have, because events in the *autos* are happening for the first time, recounting the history of Humankind.

The *attentional-gate mode* proposed by Zakay and Block (1997) relies on the suggested existence of an internal clock device made of a pacemaker emitting pulses. The more the attention is directed to measuring time, the more pulses are perceived, and the more extended the perception of the duration. Characters portraying a negative behavior do not pay attention to the time flow but to other non-recommended tasks. Cognitive load significantly regulates prospective timing (Block et al., 2010), which explains why this “vain entertainment” is fleeting, as these characters are busy enjoying themselves. Coincidentally, making long-term plans or delaying gratification are processes regulated by the prefrontal cortex, an area that could be considered defective in negative characters, based on their poor choices within the realm of the plot.

This reference to attention is crucial, given that scholars such as Glicksohn (2001) ascribe the conscious awareness of the flow of time to the relationship between attention, arousal, and time perception. The more inwardly that attention flows, the slower time is perceived, as internal events seem to flow more slowly. This, when fewer subjective time units are accumulated, is a phenomenon of hypoarousal. In this sense, to Glicksohn, the experience of time greatly depends on the individual’s immersion in the external world. As attention is directed inward, the individual becomes more mindful of the own experience, and more aware of the moment. As said, paying attention to the time flow dilates its perceived length, while diverting attention from it shortens its perceived duration (Polti et al., 2018).

While *autos sacramentales* focus on future outcomes, the way they reflect on time also functions to anchor their characters in the present. Moreover, as characters fight against the world, one of the three enemies of the soul, they become closer to be in grace, and less subject to the flow of time, until the perfect union with God, where time ceases to exist.

### Everything Relies on Memory

Calderón gives significant importance to memory in his *autos^49^*, as the memory is the guardian of the rules to be followed. For instance, in Calderón de la Barca (1996b) *El indulto general* (The General Pardon), the character Guilt relates misbehavior to a lack of memory; therefore, Man’s oblivion will be remembered in Guilt’s book of remembrances^50^. The playwright also shows awareness of the effect of cognitive load in memory. Nabuco in Calderón de la Barca (2011c) *Mística y real Babilonia* blames the act of speaking for the fact that he is losing his memories.^51^ For the playwright, memory is so crucial in the correct development of personality that in Calderón de la Barca (1996a) *El cordero de Isaias* (The Lamb of Isaiah), the character Descuido (Neglect) is characterized by the lack of memory^52^. In his *autos*, memory is portrayed as a device^53^ connected to the past^54^, and Thought is the one in charge of bringing pieces of information to Memory^55^. Finally, time can modify memory, even delete it.^56^

Unsurprisingly, memory also appears in historical or biblical *autos* as the custodian of the biography of characters, such as in Calderón de la Barca (2001b) *La protestación de la fe* (The Protestation of the Faith), in which queen Christina says: “las dudas/que en él padezco lo digan/revolviendo en mi memoria/moviendo en mi fantasía/mal formado embrión de todos/los sucesos de mi vida.”^57^ Calderón also links memory to sensation, as in Calderón de la Barca (2003) *El gran mercado del mundo* (The Great Market of the World), where Gluttony talks about bringing tastes back to memory^58^, and in Calderón de la Barca (1997a) *El primer blasón de Austria* (The First Blazon of Austria), where memory is again mentioned concerning food^59^. As previously mentioned, in Western culture, gluttony symbolizes the lack of impulse control. Nevertheless, when it comes to remembering, smell has a much more critical role than taste. This preeminence is because, in the cortex evolution, olfaction kept its direct routing to the hippocampus, the seat of memory, while other senses were re-routed through intermediary connections (Zhou et al., 2021). There is evidence for a relative time-based code in the olfactory bulb (Haddad et al., 2013).

The playwright also places memory in the role of the retriever of that which is essential^60^. The clearest example of this is the Ley of Gracia (Law of Grace) in Calderón de la Barca (2021) *El gran teatro del mundo* (The Great Theater of the World), who acts as the lines prompter and is constantly reminding other actors of the need to behave properly. This understanding of memory as the retriever reflects how memory performs better when based on cues rather than on voluntary retrieval (Staugaard and Berntsen, 2019). In his autos, we encounter several characters providing cues that help the protagonists to choose the right path. At a still higher level, this repetition also explains why messages are uttered frequently through different means, i.e., in weekly mass and an annual *auto sacramental* show. Calderón also recognizes the volitive aspect of memory, as recollection can also be voluntary, as with the characters of Sinagoga and Judaísmo state in Calderón de la Barca (2005a) *El orden de Melquisedec^61^* (The Order of Melchizedek) or Entendimiento in Calderón de la Barca (2006) *La divina Filotea^62^* (The Divine Philothea).

Memories exist in time and space. Our memories are processed mainly by the hippocampus, in the temporal lobe. Episodes are placed in a timeline on which our self-identity relies. To remember memories related to our lives, episodic memories, we need to travel back in space and time (Tulving, 2002). The neural circuits used to navigate distances are analogous to the ones used to recall episodic memories. Navigation, which is processed in various brain areas by gathering information from different systems and perceptual mechanisms, operates with motions stored sequentially (Park et al., 2018). Likewise, biographic memories also get stored sequentially, creating the perception of the flow of time. This sequentialization of movements, places, and times all intertwined in the way we process it, are an important aspect of the metaphor with which Calderón constructs his plays. The past, the present, and the future are located not only in the timeline of the plot but they are also represented in different or transmuted locations. The garden expulsion in Calderón de la Barca (2000b) *El veneno y la triaca* is an example of this. When the Princess recovers grace, she disappears through the upper part of the stage, in a ship in the company of Christ.

Location is significant to memory retrieval. Themed-evoked memories prompt a higher number of overall details; however, location-prompted memories deliver a higher concentration of episodic detail. In other words, thematic information articulates multiple memories, but spatial information brings back specific episodic content from past events (Sheldon and Chu, 2017). Therefore, the expulsion from the garden implies a change of locations for the protagonists, which is translated on stage by a changing of the set. This last trait is genuinely remarkable in *autos sacramentales*, as they were performed in the streets, on top of two to four different carts, signaling different times and places. “In Madrid these plays were acted on platform stages, or on carts used as stages, and the scenery for them was built on other carts which were wheeled into position behind the stage or at its sides” (Shergold and Varey, 1964). When acted the change of location happens not only in the dramatic space but also in the physical space.

Navigation and time being related to memory’s neural processes is also consistent with the metaphor present in the doctrinal language: human characters, the ones getting “lost,” have defective memories. For instance, distracting factors can cause memory to forget crucial things, as Beauty does in Calderón de la Barca (2005b) *El verdadero Dios pan^63^*, but this is more a problem of attention. In Calderón’s texts, memory and attention are sometimes indistinguishable, which mirrors how working memory and attention share the same neural mechanisms in the prefrontal cortex. Acting on sensory information is usually considered attention while acting on inner thoughts tends to fall within the realm of memory. This might the reason why the world, which is in charge of diverting attention from interior contemplation, is considered one of the three enemies of the soul, along with flesh, and the devil.

In the *autos*, divine characters have infallible memories. In the case of God, ever-present in time and space, although Calderón will sometimes say that he never forgets about his creatures, his is a matter more related to attention than to memory itself. God, or God proxies, also tell in several *autos* the whole story of creation until the moment in which humankind starts to exist and begins to undergo trials.

The Devil also has a perfect and exact memory^64^ which contributes to the dramatic structure of the plays. The Devil is a recurrent instigator of the plot, which he sets in motion by lying a trap for the Human Nature, and Calderón places particular emphasis on justifying his motives for behaving like he does. The *autos* often contain long series of verses in which the Devil explains his biography in extreme detail: the circumstances in which he was expelled from heaven, his jealousy and envy regarding humankind (a very cherished motive for Calderón), and how he plans to take revenge. Here Calderón illustrates how individuals tend to give much more detailed descriptions of their memories than they do of our imaginations. His biographical accounts are full of trauma, which is consistent with how memories filled with intense emotions are more easily fixed (LaBar and Cabeza, 2006). It is also consistent with the role of negative emotions in enhancing the mnemonic precision of memories. This increase in memory precision is also associated with elevated subjective feelings of the vividness of remembering and metacognitive sensitivity (Xie and Zhang, 2017).

A last point regarding time and memory is that in the allegory of encapsulating humankind’s history in just 2 h of scenic time, a very compressed time, is neither relevant nor conducive to showing characters deducing time lengths retrospectively. This is particularly true when the whole genre is devoted to calibrating moral choices for future outcomes. This is the same reason that while enjoying oneself time paradoxically “flies,” the opposite happens when recalling how much joyful time has elapsed, which seems elongated. Likewise, those who have experienced more novelty in their lives perceive that they have lived for a longer time. This is in contrast, to those with routinary experiences, who perceive their lives to be shorter. This dilating phenomenon can depend on the stamping of more brain time encodings and on a neural coding of episodic memory, in which codes are generated about what, where and when, in the entorhinal cortex. Memories with more novelty in these three parameters are more likely to be remembered, because all parameters combined will have created more time codes (Sugar, 2019). Maybe, an eternity in hell means the constant recurrence of suffering, while eternity in heaven might mean complete attention to time or a never-ending succession of novelty.

### Decision-Making

Withing Calderón’s *autos sacramentales*, every second, every instant counts toward an eternity in glory or an eternity in pain, and deciding only takes a moment.^65^ One is even titled, (Calderón de la Barca, 1996d) *No hay instante sin milagro^66^* (There Is Not a Moment Without a Miracle), emphasizing the idea of the power of an instant. The playwright turns the present into a crucial moment of his *autos*’ ethos. He insists on the importance of a single moment, as it is in a single instant that decisions are made and changes happen in this theatricalized history in which time is compressed.

There is an ontological reason to remark the importance of the *instant*, as the brain takes only milliseconds to process perceptions and make decisions. This proper instant connects with the idea of *kairos*, from Greek philosophy, with the meaning of “the right time.” This right time represents a moment of undetermined duration in which something important happens and that in Christian theology is related to “the time of God.” Therefore, time has not only a quantitative value but also a qualitative value. What gives value to human life is its temporariness, thus, the importance of the reminder of death. The memory of death is a must in a life well lived in this context (repeated consistently in these texts) as a registered negative outcome. For this reason, its remembrance always brings sadness to men^67^.

Each moment is critical because in a moment, we can take one path or another in the journey of life, integrated into the history of Christian salvation. To make the correct decision, our characters, as humans, are forced to practice mental traveling, relying on memory and imagination because decision-making is nothing but the link between memory and future actions (Fellows, 2018). According to the Teleofunctional Theory of Representation proposed by Millikan (1984), this happens because “temporal representation becomes explicit when a creature is capable of representing future goals separately from the specific routes to achieve them. The consideration of alternate possible actions requires a representation of the future as temporally distinct from the present” (Droege, 2009). This representation of the future befits these plays’ moral teleology and the teleology of voluntary decisions. Likewise, decisions are heavily influenced by the perceived duration of the various options, the possible delays for the outcomes, and the time constraints for making a choice.

In Calderón’s *autos*, the problem of Humankind committing the original sin is a problem caused by the lack of memory, the inability to travel back in time to gather information from past experiences. It is a problem of knowledge/ignorance. The characters are ignorant of the consequences, as they have never experienced them. It is also a problem of the impossibility of “acting for the future,” because in the sanctifying grace, there is no future, being time suspended as we have seen.

*Autos sacramentales* usually show characters with poor *affective forecasting*, the prediction of one’s future emotions (Wilson and Gilbert, 2003), and with a high *temporal discounting*, the preference of small immediate rewards over delayed increased rewards. These characters choose fleeting pleasure over eternity next to God. Regarding the latter, one of the main differences between the Baroque period and contemporary societies is the process of secularization. While the remembrance of death in the 17th century could provoke a decrease of temporal discounting, psychological research has shown that today, the thought of death produces the opposite effect, increasing the preference for immediate rewards (Story et al., 2015). Neuroimaging has also revealed that thinking of death influences self-referential processing (Chen et al., 2019) and empathy (Li et al., 2015), which may be another reason for death’s presence in this type of literature.

Regarding poor affective forecasting, humans provide more accurate affective forecasts based on experience rather than descriptions. In Calderón’s *autos*, protagonists do not heed when their mentors warn them about the adverse outcomes of wrong decisions. It is experience with negative consequences what most accurately helps predict one’s future emotions (Fu et al., 2018), but these characters do not have that experience. Without memory, projecting ourselves into the future becomes a challenging task (Addis et al., 2007).

As previously mentioned, the hippocampus is the epicenter of long-term memory, but it is also essential to short-term and spatial memory and real-time decision-making. When surveilling the environment searching for navigational cues, the hippocampus becomes active, combining short-term memories and visual information to make decisions on the spot. In other words, the hippocampus uses memory to direct sight, allowing the visual system to reevaluate the environment and create connections between related visual stimuli. These short-term memories of visual stimuli, once connected by the hippocampus, can help inform decisions and can become long-term memories (Kragel et al., 2021).

The connection of these brain processes might shine light on the theater’s inner workings, as movement on stage, the use of visual stimuli, and decision-making are indispensable in dramatic art (drama comes from the Greek *dráô*, “to act, to take action, to achieve.”) Finally, decision-making cannot be separated from the learning process, as both processes share an underlying brain mechanism called the cortico-basal ganglia-thalamic loops (Dunovan et al., 2019). Let us not forget *autos sacramentales* are, besides theatrical shows, pedagogical artifacts.

## Conclusion

As we have seen, Calderón’s *autos sacramentales* deal with the concept of time from many different perspectives: how individuals evolve in it, how it relates to transcendent realities, how it flows, and how it shapes the possible salvation or damnation of humankind. Just as our brains order events sequentially when the hippocampus registers the sequential activity of neuronal assemblies and their internally defined rate of change, the playwright uses the also sequential setting of Christian historiography to pose his plots. Each stage of the plot follows the previous one with defined shifts and immediate causal relations that force changes in circumstances. Because of the brain’s motor decoding predisposition, continuous events are more easily linked as cause-effect. This continuity also influences the use of object metaphors which facilitate the brain in establishing that relation. The importance of different locations for different moments also reflects how time computation is intimately linked to space in our brain processing, specifically in the parietal cortex, as learning about the environment happens through motor actions. This sequential structure insists on the importance of being aware of the flowing of time, which can dilate or compress its length estimation based on the focus of attention. It also emphasizes the one-directional flow of time, and how human perception processes it due to the entropy also registered in neural states. In theater, entropy is related to the break of order, and in the human brain, with creativity and divergent thinking.

The playwright portrays how being in time (mortal or eternal) defines the different types of persons, human or divine, and shows how this temporal duality is present in Christ, the link between both realms. Mindfulness of time identifies positive characters, while external attention characterizes negative characters, whose time seems to flit away due to cognitive overload. Equally, the villains fail to see long-term consequences and err in their choices, which relates to the performance of the prefrontal cortex. On the other hand, time measurement linked to interoception, especially related to the digestive apparatus, allows Calderón to articulate the existence of an inner time clock based on internal sensations when characters express hunger.

The computing of natural rythms, infradian, circadian, and ultradian, also shapes the dramatic structure and allows different metaphors about time behavior. The day works as time measurement, with a clear beginning and end (the change of light perceived by our organism), and this is turned into the metaphors of birth and death. Another remarkable feature is the use of seasonal change perception, ruled by neuronal oscillations of the suprachiasmatic nucleus, which, combined with astrocytes, encodes, and distributes circadian and seasonal information. The portrayal of these cycles in these plays supports the Aristotelian bond between change and time. When seasons do not change in these plays, time does not flow, revealing a reality different from that of humankind.

Finally, because we can ultimately call Calderón’s *autos sacramentales* the study of choice, the playwright shows us how mental time-traveling is *conditio sine qua non* to make the right decision. The ability to recall important information and project probable future outcomes is crucial to reach salvation. The connection between Western philosophy along the centuries and cognitive science and neuroscience sheds light on how the Spanish baroque experience saw itself through its members and how Calderón, a son of his time, conceived human nature.

## Data Availability Statement

The raw data supporting the conclusions of this article will be made available by the authors, without undue reservation.

## Author Contributions

The author confirms being the sole contributor of this work and has approved it for publication.

## Conflict of Interest

The author declares that the research was conducted in the absence of any commercial or financial relationships that could be construed as a potential conflict of interest.

## Publisher’s Note

All claims expressed in this article are solely those of the authors and do not necessarily represent those of their affiliated organizations, or those of the publisher, the editors and the reviewers. Any product that may be evaluated in this article, or claim that may be made by its manufacturer, is not guaranteed or endorsed by the publisher.

## Footnotes

1 Churchill (1974, 6897).

2 This idea can be found in several Aristotle’s works: Phys. 1943 21, 1993 17; De Mundo 396b12; Poetics 14473 14-17, 1448b4-24.

3 Wittmann, 2014.

4 Other cultures deal with time differently. For instance, the Amondawa (Tupi Kawahib) in the Amazon do not conceive time as something in which events occur (Sinha).

5 In June of 2021, *The Journal of Neuroscience* published a study entitled “Human hippocampal neurons track moments in a sequence of events,” directed by Reddy et al. (2021) from the Brain and Cognition Research Center (CerCo) in France. This study identifies a group of neurons in the hippocampus that encode temporal information. According to this research, these “time cells” piece together temporally different elements of an experience into a coherent memory, which allows the existence of episodic memory.

6 For a review on dedicated and intrinsic models see Ivry and Schlerf (2008), “Dedicated and intrinsic models of time perception.”

7 Seize the day, time flies, remember you must die.

8 For further discussion on this matter see Moncunill Bernet (2012), “El optimismo antropológico calderoniano en sus autos sacramentales.”

9 “Sermones/puestos en verso, en idea/representables cuestiones/de la sacra Teología,/que no alcanzan mis razones/a explicar ni comprender.” “Sermons in verse, Sacred Theology topics represented as a performance, that my intellect cannot understand nor explain.” *Loa* of *La Segunda Esposa.* (Valbuena Prat 427). N.B.: All translations are mine.

10 Calderón de la Barca (1995b) *El año santo de Roma* (The Holy Year in Rome) states*:* “lo que fue, y es, será/reducido a un argumento” (v. 491–492). What was, is, and will be, reduced to a single plot.

11 *La lucha entre el bien el mal no tiene fechas y la capacidad redentora de la eucaristía es eterna. Quizás el auto sea el género dramático que más sistemáticamente considera el tiempo* sub specie aeternitatis.

12 Time is a character in Calderón de la Barca (2004a) *El día mayor de los días* and Calderón de la Barca (1996c) *La nave del mercader.*

13 Calderón de la Barca (1996c) *La nave del mercader* (vv. 2245–2246).

14 Calderón de la Barca (2004b) *Los encantos de la culpa* (The Charms of Guilt), Calderón de la Barca (2001a) *El socorro general* (The General Relief), Calderón de la Barca (1987, 2011b) *La iglesia sitiada* (The Besieged Church), or Calderón de la Barca (1987, 2007) *El jardín de Falerina* (The Garden of Falerina).

15 This idea was further developed by Idealist German Philosophers, such as Hegel and Schelling.

16 This is the conventional division of the History of Salvation. The period of the Natural Law goes from the Fall of Adam to Moses, in which the moral guidance comes from the precepts written by God in every human being. The Written Law encompasses the time from Moses to Jesus, and it refers to the Pentateuch. Finally, the Law of Grace goes from the Advent of Christ to the Final Judgment: the moral guidance in this period comes from Jesus’ teachings and Church Precepts.

17 A slightly exceptional case are the narratives in installments in which later installments overlook changes that occurred in earlier installment, a common device in TV shows.

18 St. Augustine (2021a) *City of God*, Book XII-13.

19 In the universe, there was a highly ordered “beginning” point: the Big Bang. The entropy of the universe at the Big Bang was extremely small. Because of the second law, and because the universe is an isolated system (by definition), the entropy of the universe has been increasing ever since the Big Bang. The Big Bang breaks the time-symmetry of the universe resulting in the arrow of time that we experience, with the past pointing toward the Big Bang (low entropy) and the future pointing away from it (higher entropy). In a universe without a Big Bang, or in a universe that has reached a state of maximum entropy, there would be no arrow of time concept. Whether time itself would still exist in such a universe is an open question in physics.

20 However, shorter intervals do not always lead to stronger causal relationships (Bramley et al., 2018).

21 St. Augustine (2021a) explains in *City of God* his doctrine about sacraments. In Book X he says that sacraments are a demonstrative sign of the present invisible sacred realities.

22 Calderón de la Barca (1995a) *Andrómeda y Perseo* (Andromeda and Perseus), Calderón de la Barca (1996c) *La nave del mercader* (The Ship of the Merchant), (Calderón de la Barca, 1997c) *Sueños hay que verdad son* (Some Dreams are True), Calderón de la Barca (2004a) *El día mayor de los días* (The Most Important Day), Calderón de la Barca (2011a) *El pleito matrimonial del cuerpo y el alma* (The Divorce of Body and Soul), Calderón de la Barca (2011c) *Mística y Real Babilonia* (Mystic and Royal Babylon), Calderón de la Barca (2013a) *La siembra del señor* (The Sowing of the Lord), Calderón de la Barca (2014a) *El divino cazador* (The Divine Hunter), and Calderón de la Barca (2014b) *La cura y la enfermedad* (The Cure and the Disease).

23 “Aire […] cuerda con que concertado/andaba siempre acá dentro/del reloj del corazón/el volante del aliento;” (vv. 101–105).

24 For example, his plays Calderón de la Barca (2000a) *Basta callar* (The Silence is Enough) and Calderón de la Barca (1983) *Entremés del reloj y los genios de la venta* (Interlude of the clock).

25 “*una incompatibilidad fundamental de dos conceptos del tiempo: (1) el tiempo del campo, de las pasiones humanas, y de la naturaleza, medido por los ciclos del sol y (2) el tiempo urbano, el del rey y la corte, medidos por relojes que deben sincronizarse con el reloj de palacio*” (203).

26 Lodovico Castelvetro, an Artistotle interpreter, who is to a great extent responsible of the philosopher’s units Renaissance codification, was strict enough to limit the *day* to *12 h*, as for him it was not possible that the audience would accept the idea that the plot takes longer than the time passing that they directly experience (Urban, 1966, 242).

27 Calderón chooses in some of his *autos* to present day and night as characters.

28 So much so that Calderón uses it to give title to one of his *autos*: Calderón de la Barca (2004a) *El día mayor de los días*.

29 Vv. 707–710, 809–810, 895–897.

30 The cyclical essence is also present in many aspects of the Christian practice. First, and related to this theater practice: *autos sacramentales* were written to be performed every year on the day of Corpus Christi, around June, following a pattern of annual repetition. In the same way, the sacrifice of Christ is relived in each celebration of the mass, which somehow contributes to repetition of the present of such sacrifice. The religious and secular rituals dealing with the question of salvation work as a routine used to diminish spiritual anxiety.

31 Calderón de la Barca (1999) *El Divino Orfeo* (The Divine Orpheus, vv. 1259–1260).

32 For example: the soul “En el reino del espanto 1055 cautiva estaì con eterno dolor”; The soul is captive in the horror kingdom, suffering eternal pain. Calderón de la Barca (1999) *El Divino Orfeo* (vv. 1055–1056). “Eterno castigo”; eternal punishment. *La vida es sueño*, segunda versión (Life is a Dream, Second Versión, vv. 305).

33 While in math the possibility exists for the countably infinite and the uncountably infinite, in philosophy infinite is often understood as “exceeding measurement or calculation” and “‘immeasurably great in extent (or duration, or some other respect).’ This strict, non-mathematical sense is often applied to God and divine attributes, and to space, time and the universe.” *Stanford Encyclopedia of Philosophy.*

34 The translation is mine.

35 “The clock announces mealtime when I am not hungry, and when I am hungry, I have to wait until the clock rings for mealtime. Fools, eat whenever you are hungry, sleep whenever you are sleepy, because we cannot allow our entrails to be the inner workings of the clock” (vv. 407–412).

36 Plato divides the soul in three parts, and the stomach is related to the concupiscible soul, which is in charge of basic vital operations.

37 In *Physics*, Aristotle (2021b) defines time as the measure of movement (IV-10), and in Aristotle (2021a) *Metaphysics*, he defines eternity as immobility (*primum muvens*, XII-7). Although he does not identify time with movement, he states that there cannot be time without movement, as both are perceived together. At the same time, he wonders if time can exist without a soul who perceives it, introducing the concept of subjective time, which also is paramount in these plays.

38 “For I the Lord do not change; therefore you, O children of Jacob, have not perished” (Mal. 3:6, NRSV).

39 One of the most well-known debates about the role of human free will and divine grace regarding salvation was the *Congregatio de Auxiliis*, held in the last years of the 16th century in places like Spain and Rome. It ended without reaching a conclusion about which was the real extent of human free will on the path to salvation.

40 *Confessions* 11.17.

41 “There are in me, united, two natures, the human and the divine, and I signify myself in them both: my face is eternal, my feet plants deciduous” (vv. 73–78).

42 Calderón de la Barca (2008) *Tu prójimo como a ti*, segunda versión (Your Neighbor as Yourself, second version)

Culpa: Ya habeìis visto en las ideas
que fantaìsticas os finjo
cómo es el hombre viador,
cómo es la vida camino^42^, […] (v. 363–366).

“You have seen in the fantastic ideas that I create for you, how Man is a traveler, how life is path” (v. 363–366).

43 And other linguists such as Traugott (1978).

44 Brief, deciduous, ephemeral, perishable, term.

45 At once, quickly, briskly, late, rapid, fast, slow, swift.

46 To approach, to run, to hurry up.

47 We can find examples of this in Calderón de la Barca (2006) *La divina Filotea* and Calderón de la Barca (2013b) *No hay más fortuna que Dios* (There Is Not Better Fortune Than God).

48 Only God has practical knowledge to know the world exactly as it is; humans only have speculative knowledge. These terms are discussed by Aquinas (1947) in *Summa Theologiae*, I, q. 14: God’s knowledge.

49 In Calderón de la Barca (2011a) *El pleito matrimonial del cuerpo y el alma*, Body decides to abandon the Eucharist banquet. Afterward positive characters remind him of the mysteries behind the sacred bread. When they speak, the Memory walks toward them. Memory is the only character in movement in this scene, and only stops when Sin denies the presence of Christ’s Body and Blood in the Eucharist. After just a moment, Memory resumes walking toward the speaking characters. This movement represents how Memory is the one in charge of providing the images of knowledge. Later, Body recriminates: “>Cuándo/Memoria, has de descansar?” (Memory, when will you rest?) (vv. 659–662).

50 “Sea/memoria de sus olvidos/el libro de mis acuerdos.” “May the book of my remembrances be the memory of his forgetfulness,” (vv. 895–897). The same idea of Guilt, or the Devil, keeping a registry of human errors appears in Calderón de la Barca (1997b) *La inmunidad del Sagrado* (The Immunity of the Sacred), when Lucero says: “>Qué error mortal/hay, que en memoria no esté/mía? (What lethal mistake exists that is not in my memory?, vv. 413–415, Calderón de la Barca, 1997b).

51 “Yo vi… no sé qué vi, que no me acuerdo,/con el discurso la memoria pierdo,” “I saw… I do not know what I saw, I do not remember, because when I am speaking I forget” (vv. 68–684).

52 Vv 898–700.

53 Books are also memory devices for Calderón as we see in Calderón de la Barca (2005c) *La vacante general* (The General Job Opening, vv. 854–857).

54 Time refers to memory this way (v. 402).

55 Calderón de la Barca (2004a) *El día mayor de los días* (vv. 449–452).

56 As it is said in Calderón de la Barca (2009) *El árbol de mejor fruto* (The Tree with the Best Fruit): “ya que con el tiempo/tan de memoria se pierde,” “with time, memory is lost,” vv. 875–876).

57 “The doubts in my chest can say it, by stirring my memory, shaking in my fantasy, the deformed embryo born from all my life events” (vv. 933–938).

58 “Pinturas, que pintadas/todas mis glorias son imaginadas/porque cuanto apetece/el hombre el Apetito se le ofrece,/trayendo a su memoria los empleos/de gustos, de manjares y deseos,” “Paintings, because all my imagined glories are painted, because Appetite serves Man what he wants, bringing to his memory tastes, delicacies, and desires” (vv. 893–897).

59 “Aquesto es mental historia,/engañar el pensamiento,/llenar las tripas de viento,/y comida de memoria,” “This is a mental history, to fool the thought, fill the entrails with air and remembered food.” (vv. 389–392).

60 Calderón de la Barca (1996b) *El indulto general*, we see how memory plays an important role in the love that Christ feels for the human soul: “Tan en mi memoria estás/desde el punto que te vi,/que a mi ser tu ser uní, con tan suave lazo fuerte/que me ofreceré a la muerte,/por darte la vida a ti” “you are constantly present in my memory since the first moment I saw you, and we are both united with such a soft and strong knot, that I will offer myself to death in exchange for your life” (vv. 662–667).

61 Vv0.301, 303, 471, 1660.

62 Vv. 832.

63 “Pues toda hermosa eres,/no oigas, que siempre ha sido/triunfo de la hermosura/de otras memorias componer olvidos,” “because you are beautiful, do not listen, because it is beauty’s triumph to erase other memories” (vv. 1279–1282).

64 “Y es imposible que llegue/yo a olvidar, porque no olvidan/espíritus lo que aprenden,” “It is imposible for me to forget, because spirits do not forget what they learn” Calderón de la Barca (2000b) *El veneno y la triaca* (vv.625–627).

65 Ten seconds before reaching consciousness (Soon et al., 2008).

66 In this *auto*, Dimas, the good thief, affirms the importance of repentance and reminds the audience that a miracle, like anything else, can happen in an instant.

67 Death acknowledges this sorrow in Calderón de la Barca (2005d) *Lo que va del hombre a Dios* (The Distance Between Man and God)*:* “En los humanos pesares/soy quien tiene tanto imperio,/que con la sola memoria/al más alegre entristezco,” “Over the human sorrows/I have such a dominion/that by only recalling me/the happiest one becomes sad” (vv. 487–490).
